# Cathelicidins: Immunomodulatory Antimicrobials

**DOI:** 10.3390/vaccines6030063

**Published:** 2018-09-14

**Authors:** Roel M. van Harten, Esther van Woudenbergh, Albert van Dijk, Henk P. Haagsman

**Affiliations:** Division Molecular Host Defence, Dept. Infectious diseases and Immunology, Faculty of Veterinary Medicine, Utrecht University, Yalelaan 1, 3584 CL Utrecht, The Netherlands; R.M.vanHarten@uu.nl (R.H.); e.vanwoudenbergh@students.uu.nl (E.W.); a.vandijk1@uu.nl (A.D.)

**Keywords:** cathelicidins, host defense peptides, antimicrobials, immunomodulation, chemotaxis, adjuvants

## Abstract

Cathelicidins are host defense peptides with antimicrobial and immunomodulatory functions. These effector molecules of the innate immune system of many vertebrates are diverse in their amino acid sequence but share physicochemical characteristics like positive charge and amphipathicity. Besides being antimicrobial, cathelicidins have a wide variety in immunomodulatory functions, both boosting and inhibiting inflammation, directing chemotaxis, and effecting cell differentiation, primarily towards type 1 immune responses. In this review, we will examine the biology and various functions of cathelicidins, focusing on putting in vitro results in the context of in vivo situations. The pro-inflammatory and anti-inflammatory functions are highlighted, as well both direct and indirect effects on chemotaxis and cell differentiation. Additionally, we will discuss the potential and limitations of using cathelicidins as immunomodulatory or antimicrobial drugs.

## 1. Introduction

The rise of antimicrobial resistance requires us to think differently about microbial infections. Antibiotic resistance is a growing global problem from both an economical and a societal point of view [1]. Combating this problem is one of the major challenges for health care in the 21st century. A solution will likely require global approaches the problems surrounding antibiotic resistance, with both scientific and legislative contributions, and will include efforts from agricultural and pharmaceutical industries, particularly in developing countries.

Additionally, new drugs are required to maintain a suitably large pool of treatment options against bacteria resistant against conventional drugs. In the drug discovery field, conventional target-based approaches to discovering new antibiotics have failed to produce new classes of antibiotics thus far [2]. Future medicine will require intensive resistance monitoring [3] and inverse selection against resistance [4], while drug discovery will have to look for innovative methods to discover new classes of antimicrobials [5].

Furthermore, alternative strategies other than looking for classical antibiotics should be considered. For example, promising results have been obtained by using directly lytic antibodies [6,7]. These antibodies have low immunogenic properties and are extremely target-specific (i.e. leaving no impact on the host microbiome), but their development has been challenging [8]. Accruing evidence of immunological memory in the innate immune system could also lead to potential new therapies that boost the host’s non-specific defense to kill pathogens rather than targeting the pathogen itself [9,10,11,12].

Host defense peptides (HDPs) could be used as potential immunomodulatory molecules as another way to treat microbial infections. As part of their innate immune system, most animals produce a wide variety of HDPs, which possess both antimicrobial and immunomodulatory properties [13,14,15]. Their pleiotropic expression and targets, structural diversity, and reactivity across species make them interesting candidates for novel therapies or supplementation for existing treatments. 

Host defense peptides have various genetic and structural classifications. The main classes are cathelicidins and defensins. Cathelicidins are characterized by a cathelin-like domain and defensins are defined by their intramolecular disulfide bonds [16,17]. In addition to these, other smaller classes of HDPs can be distinguished [16,17].

In this review, we will focus on cathelicidins, examining their various immunomodulatory functions and their potential novel use for therapies. 

## 2. Structural Aspects

Cathelicidins have been characterized in many phyla of animals, including mammals, birds, reptiles, amphibians, and some fishes [18]. The number of different functional genes encoding cathelicidins differs greatly between animals, from only one gene in humans, mice, rats, dogs, and guinea pigs to several genes in pigs, cows, chickens, rabbits, horses, goats, and sheep, with pigs having 11 cathelicidin genes [19,20,21]. It is not known whether there is a functional implication is for these species-specific cathelicidin repertoires.

Cathelicidins are encoded by genes consisting of 4 exons [20]. Exon 1 encodes a 29 or 30 amino acid (AA) signal peptide. Exon 2 and 3 encode the conserved cathelin domain of 99 to 114 AAs [22]. Exon 4 encodes the mature peptide of 12 to 100 AAs, and possesses antimicrobial and immunomodulatory properties [23]. Cathelicidins are produced as pre-pro-peptides and stored inside granules. Upon activation of the cell, they are secreted, and the N-terminal pro-domain which includes the cathelin domain is cleaved off to form the mature, biologically active peptide [20]. Even though the cathelin domain is very conserved between species, the mature peptides are very diverse. Additionally, cleavage may generate products of different lengths. The only human cathelicidin gene, hCAP-18, for example, has been shown to yield at least three different mature peptides, of which LL-37 is the most commonly studied [24,25]. However, even without much homology at the sequence level, the mature cathelicidin peptides share certain physicochemical properties. Firstly, they are highly cationic with a charge that can vary between +4 and +13 in physiological conditions [26]. Secondly, many cathelicidins have an unstructured conformation in an aqueous environment but can adopt an α-helical structure in the presence of a membrane [27,28,29]. Other mature cathelicidin peptides may adopt β-sheet structures, such as the cyclic protegrins with intrachain disulfide bonds. Some linear cathelicidin peptides are enriched in certain amino acids, e.g. tryptophan for bovine indolicidin, or proline and arginine for porcine PR-39 [22]. 

These commonalities are the main attributes resulting in their antimicrobial activity: the positive charge interacts with negatively charged bacterial membranes, while the hydrophobic residues can perturb the membrane and cause cell death. In contrast, the immunomodulatory nature of cathelicidins is usually more based on stereospecific interactions with a variety of receptors, depending on the cathelicidin [21]. However, in some cases the all-D enantiomer can still cause receptor activation, indicating a binding mechanism that is probably not due to interactions with a specific three-dimensional structure, as the naturally occurring peptide would be its mirror image [30].

Cathelicidins are constitutively expressed at low levels in epithelial cells, mucosal surfaces, and the skin, and are highly released in response to infections by particularly granulocytes and mononuclear phagocytes. For example, the average serum concentration of the human cathelicidin LL-37 is 1.18 µg/mL in serum of healthy individuals [31], but the local concentration upon neutrophil degranulation can be much larger [32]. For instance, LL-37 is present in broncho-alveolar lavage (BAL) at 5 µg/mL in healthy individuals but can be up-regulated to 30 µg/mL in BAL of cystic fibrosis patients [31,33,34]. The presence of LL-37 in BAL is mostly due to neutrophil degranulation. This means that a gradient of cathelicidins will surround activated leukocytes at a site of infection.

Besides infection, there are other mechanisms regulating cathelicidin levels. For example, in vitro stimulation of epithelial cells and monocytes with the active form of vitamin D causes an increase in LL-37 levels [35,36], and exogenous vitamin D administration can lead to restoration of microbe-suppressed levels of endogenous cathelicidins [37]. 

Based on their biochemical similarities, de novo design or adaptation of natural cathelicidins can be a source of novel drug candidates. Iteratively applied point substitutions, scrambling, and deletions of bovine cathelicidin bactenecin-2 led to an optimized immunomodulatory peptide IDR1018, which showed antimicrobial and anti-biofilm activity [38], suppression of lipopolysaccharide (LPS)-induced TNFα production by human peripheral blood mononuclear cells (PBMCs) [27], differentiation of M1/M2 intermediate macrophages [39], and protection from malaria in a preclinical mouse model [40]. 

More recently, computational approaches combining characteristic features of length, charge, and hydrophobicity has led to discovery of protein subdomains which could have the same functions as natural cationic HDPs [41,42,43]. For example, a region in the poorly characterized human protein 11-beta-hydroxysteroid dehydrogenase type 3 (HSD 3) showed many HDP-like characteristics, including anti-biofilm and LPS-neutralizing effects [42]. 

Although de novo design of a new, functional cathelicidin is complicated, these results have shown that new HDP-like drug candidates can be synthesized, and that optimization of existing HDPs for specific purposes is possible.

## 3. Biological Functions of Cathelicidins

**Figure 1:**
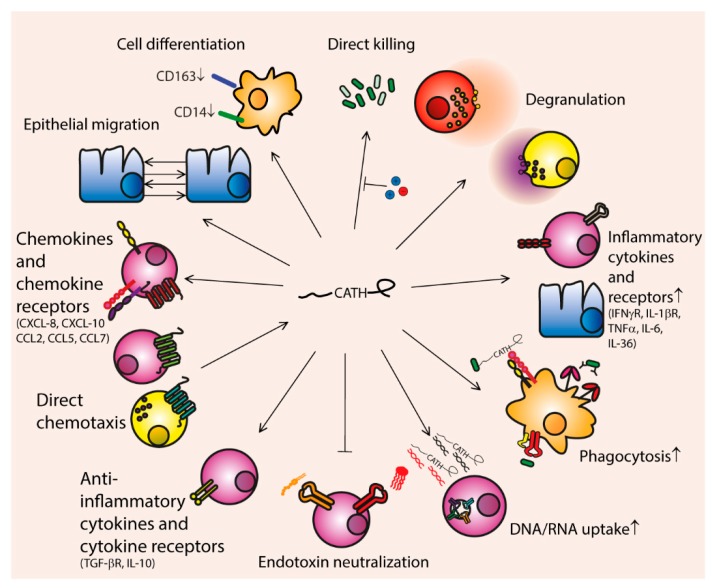
Summary of cathelicidin functions. Cathelicidins have direct killing activity against (among other pathogens) both Gram-positive and Gram-negative bacteria, but this activity is inhibited by the presence of salts. They can induce degranulation of neutrophils and mast cells, induce upregulation of inflammatory cytokines and cytokine receptors, enhance phagocytosis by opsonizing bacteria and upregulating bacterial recognition receptors, and enhance DNA/RNA uptake, thereby boosting intracellular toll-like receptor (TLR) signaling. Furthermore, they can inhibit endotoxin mediated activation of TLR2 and 4 by binding to lipoteichoic acid (LTA) and liposaccharide (LPS), respectively, and directly induce upregulation of anti-inflammatory molecules IL-10 and TGF-βR. They are directly chemotactic for mast cells through the MrgX2 receptor, and for other cells through the FPRL1 receptor; but can also induce production of a variety of chemokines and chemokine receptors, including CCL2, CCL5, CCL7, CXCL8 (IL-8) and CXCL10. They can also induce migration of epithelial cells and thereby influence wound healing. Finally, cathelicidins can influence differentiation of cells, among others by polarizing macrophages to an inflammatory phenotype (M1).

Cathelicidins are directly antimicrobial for many pathogens, including both Gram-positive and Gram-negative bacteria, fungi, parasites, and enveloped viruses in vitro (Figure 1) [28,44,45,46,47,48]. Cationic cathelicidins can bind and disrupt negatively charged membranes, leading to cell death. These peptides can also cross membranes and target intracellular processes like RNA and DNA synthesis, impair functions of enzymes and chaperones, and can stimulate protein degradation [15,20].

However, the bactericidal effects of most cathelicidins are impaired under physiological circumstances by high salt concentrations, sugars, and other host or microbial factors. Salts, glycosaminoglycans, and bacterial DNA present in the mucus of cystic fibrosis (CF) patients bind LL-37 and impair its bactericidal abilities, even at high peptide concentrations [49]. Restoring the abnormally high salt concentration in the BAL of CF patients to normal levels can also restore LL-37’s killing capacity [50]. Some cathelicidins, such as chicken chCATH-1, and chCATH-2, porcine PMAP-36, and PR-39, lose efficacy but retain some effectivity in vitro under physiological circumstances [26,46,48].

Nevertheless, the antimicrobial effects of cathelicidins must not completely be disregarded. In vivo they are readily incorporated into neutrophil extracellular traps (NETs), where they stabilize the NET and perhaps contribute to the antimicrobial function [51,52]. LL-37, along with other cationic peptides, has been shown to protect NETs from degradation by binding DNA in the NET and shielding it from bacterial nucleases [53]. Additionally, coating indwelling medical devices such as catheters with immobilized or gel-trapped cathelicidins can prevent attachment of bacteria and formation of biofilms, which reduces the risk nosocomial of infections) [54,55]. 

Considering the inhibitory effect of physiological conditions, most cathelicidins probably do not have direct bactericidal activities as their primary function in vivo. However, they still are important for prevention of microbial infections. Chicken chCATH-1, when administered intraperitoneally at concentrations of 10 mg/kg, protects mice from lethal methicillin-resistant *Staphylococcus aureus* (MRSA) infection [56]. mCRAMP-deficient mice suffer from increased severity of streptococcal skin infections, although only to cathelicidin-susceptible streptococci. Virulent, resistant strains show no change in severity, indicating that direct killing could possibly be important here [57,58]. Additive transgenic expression of porcine cathelicidin PR-39 in normal mice ameliorates the infection’s necrotic phenotype [59]. In vivo treatment of 18-day-old fertilized chicken eggs with D-chCATH-2 protects chicks from avian *Escherichia coli* (*E. coli*) infections when they are challenged 7 days after hatching [60]. The amount of peptide still present in the organs at that time is sufficiently low to conclude that the anti-infective properties of chCATH-2 are likely derived from immunomodulation. 

These data together with the impaired bactericidal functions of cathelicidins under physiological circumstances suggest that cathelicidins rather act as immunomodulatory factors rather than as direct bactericidal agents. 

### 3.1. Degranulation

**Figure 2:**
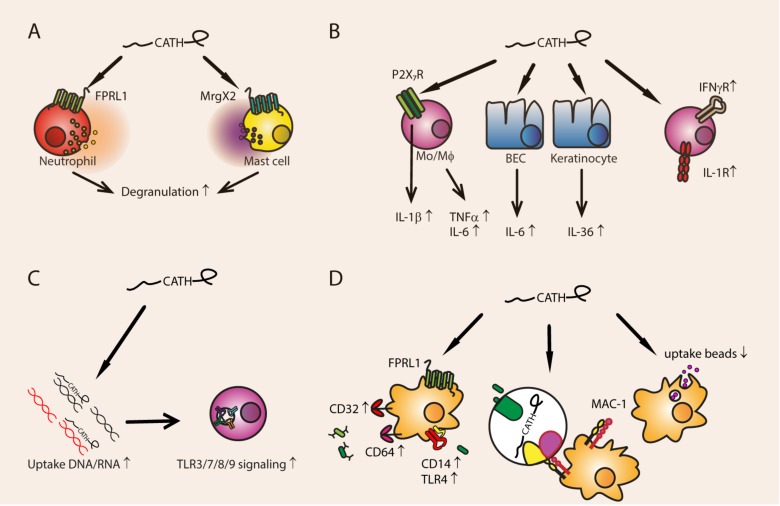
Pro-inflammatory functions of cathelicidins. (**A**): Neutrophil degranulation is stimulated through FPRL1, while mast cell degranulation is induced through MrgX2. (**B**) Cathelicidins induce production of IL-1β, TNFα, and IL-6 by monocytes/macrophages through the P2X_7_ receptor. Broncho-epithelial cells are induced to produce IL-6, and keratinocytes produce IL-36. Cathelicidins induce upregulation of IL-1R and IFNγR. (**C**) Cathelicidins bind to extracellular DNA and RNA and increase extracellular nucleotide uptake. After the cathelicidins are degraded in the endosomal compartment, TLR3, 7, 8, and 9 signaling is increased. (**D**) Through FPRL1 signaling, FcγRs CD32 and CD64 on macrophages are increased, which enhances phagocytosis of opsonized bacteria. Additionally, TLR4 and CD14 are upregulated, which increases phagocytosis of Gram-negative bacteria. Cathelicidins can also bind to pathogens and thereby opsonize directly through increased uptake by MAC-1 (CR3). In contrast, the uptake of latex beads is decreased by many cathelicidins.

Cathelicidins can stimulate degranulation of immune cells, which then release a myriad of pro-inflammatory and antimicrobial substances, including more cathelicidins (Figure 2). LL-37 stimulates mast cell degranulation via the MrgX2 receptor, which induces calcium mobilization and PI3K, AKT, ERK, and JNK activation [61,62]. This results in release of histamine and prostaglandin D2, which stimulate chemotaxis, diapedesis, and inflammation [63]. Mast cell degranulation can be induced by 10 µg/mL LL-37, which means that mast cell degranulation could be stimulated by LL-37 levels that can be present during infections [62,64]. However, LL-37-induced degranulation of a human mast cell line can be inhibited by co-administration of toll-like receptor (TLR-2) ligands [65]. This raises the question of whether LL-37 also induces degranulation in the context of an infection. 

LL-37, porcine PR-39 and PMAP-23, mouse mCRAMP, and chicken chCATH-1, chCATH-2, and chCATH-3 stimulate NO production in macrophages, but only in combination with unmethylated CpG-DNA [26,66]. LL-37 stimulates the production of reactive oxygen species (ROS) in neutrophils, most likely in a NADPH-dependent manner [67]. On the other hand, PR-39 inhibits NADPH oxidase activity, which impairs the oxidative bacterial killing of neutrophils [68]. The PR-39 mediated NADPH inhibition could be a negative feedback loop to inhibit superoxide formation and prevent tissue damage. 

Besides degranulation, 25 µg/mL LL-37 induces neutrophil extracellular trap (NET) formation via the FPRL1 receptor [69]. LL-37 and porcine PR-39 can be interwoven in the NETs [51,70]. Besides being possibly antimicrobial, these immobilized cathelicidins can prevent biofilm formation [71].

### 3.2. Pro-Inflammatory Immune Modulation

#### 3.2.1. Cytokine and Cytokine Receptor Expression

Cathelicidins regulate expression of pro-inflammatory cytokines and cytokine receptors (Figure 2). Human LL-37 [72] and bovine BMAP28 [73] upregulate TNFα production in murine macrophage cell line RAW264.7, and porcine PR-39 in porcine macrophage cell line 3D4/31 [74]. Chicken chCATH-2 induces IL-1β release from murine macrophages [75], and BMAP-28 stimulation results in activation of ERK1/2, P38 MAPK, and NF-κB, and subsequent IL-1β release, although the receptor is not known [73]. IL-1β expression is increased by LL-37 through the P2X_7_ receptor on monocytes [76]. 

P2X_7_ is a receptor for extracellular ATP which is released by damaged cells, and activation leads to inflammasome formation, processing of IL-1β and IL-18, and other downstream inflammatory processes [30,77]. LL-37 also induces the production of the inflammatory cytokine IL-36 (also of the IL-1-family) by human keratinocytes [78]. BMAP-28 and chCATH-2 increase IL-6 expression in RAW264.7 cells [26,73,79] and bronchial epithelial cells [80]. LL-37 upregulates the expression of cytokine receptors in murine macrophages, such as IL-1R and IFNγ-R [79].

It remains an open question whether induction of cytokine production happens in vivo, and if the increased levels are biologically relevant. For instance, LL-37 can increase TNFα expression in macrophages, but this effect is rather small and was only detectable at concentrations of 25 µg/mL LL-37 [26]. Here, 50 µg/mL LL-37 was needed to induce upregulation of cytokine receptors on macrophages, which are concentrations that exceed the general LL-37 concentrations present in body fluids during inflammation, although these concentrations could possibly be reached locally [79]. On the other hand, 10 to 20 µg/mL LL-37 stimulates the expression of IL-1β in monocytes, which lies within the LL-37 concentration range that could be established during infections [76]. Under non-inflamed physiological concentrations of 5 µg/mL, LL-37 is not able to increase expression of TNFα or IL-6 in human PBMCs [81]. Interestingly, LL-37 has different effects on various cell types, such as monocytes, dendritic cells (DCs), T cells, and B cells. Each of these cell types shows a different cytokine secretion pattern upon exposure to LL-37 at concentrations above 20 µg/mL [82]. 

So, the pro-inflammatory effects of cathelicidins in vitro depend on the cathelicidin used, the concentration of the peptide, and the cell type studied. Furthermore, stimulation of inflammatory cytokine production by cathelicidins in vivo may not be physiologically relevant. Although in inflammatory conditions the local concentrations of cathelicidins could possibly be high, this would be the result of inflammation rather than the cause of inflammation. Therefore, the pro-inflammatory effects of cathelicidins should be seen as amplifying or modulating but not causing inflammation directly. Additionally, many cathelicidins are cytotoxic to mammalian cells at high concentrations [83,84]. Thus, any effects (particularly inflammatory) seen at very high concentrations could also be due to cathelicidin-related cellular damage.

#### 3.2.2. Phagocytosis

Cathelicidins stimulate the uptake of pathogens via phagocytosis (Figure 2). LL-37 binds to bacteria and can simultaneously bind to complement receptor MAC-1 on monocytes and macrophages, thereby opsonizing the bacteria [85,86]. Furthermore, LL-37 and mCRAMP can indirectly enhance phagocytosis by human monocyte-derived macrophages through activating the FPRL1 receptor, which results in up-regulation of the Fcγ receptors CD32 and CD64, TLR4, and the TLR4 co-receptor CD14 [87]. This way, LL-37 and mCRAMP stimulate phagocytosis of IgG-opsonized bacteria, and non-opsonized Gram-negative bacteria. Both blood monocyte-derived macrophages and cultured microglia of mCRAMP-deficient mice indeed show reduced phagocytosis, and their bone-marrow derived macrophages have lower levels of CD14 and Fcγ-receptor expression [87,88]. 

On the other hand, a comparison of cathelicidins of various species showed that mCRAMP, K9CATH, chCATH-1, chCATH-2, and porcine PMAP-23 and PMAP-36 reduced uptake of latex beads by murine macrophages at concentrations ranging from 1.5 to 25 µg/mL, while equine eCATH-2 stimulated the uptake of latex beads [26]. LL-37, eCATH-1, eCATH-3, chCATH-3, and PR-39 did not influence phagocytosis [26]. This discrepancy between effects on phagocytosis by mCRAMP (among others) might be due to the difference between uptake of latex beads or phagocytosis of live bacteria. In contrast to beads, both the uptake and processing of live bacteria will induce immune signaling in the cell, which will cause activation of innate receptors and autocrine signaling through inflammatory cytokines [89]. Additionally, the contrast between cathelicidin effects on phagocytosis shows that the unique features of one cathelicidin cannot be generalized to others. 

#### 3.2.3. DNA- and RNA-Mediated TLR Activation

Cathelicidins can stimulate uptake of extracellular bacterial or self DNA and RNA (Figure 2). LL-37 and chCATH-2 bind to DNA released by lysed bacteria or damaged cells and increase its endosomal uptake by macrophages [72]. In the endosomal compartment, the DNA-bound cathelicidin prevents TLR9 activation until it is degraded. The interaction between the DNA and the cathelicidins is probably electrostatic. However, mCRAMP, which has an identical charge to LL-37 (+6), does not induce enhanced DNA uptake and TLR9 activation in macrophages [72]. So, while binding can be electrostatic, other mechanisms are also responsible for the increased uptake of DNA. 

LL-37 also stimulates CpG-DNA uptake and TLR9 activation in B cells and plasmacytoid DCs (pDCs), but not in T cells [90]. This effect was visible within 30 min using 6 µg/mL LL-37 in the presence of serum. This indicates that LL-37 could stimulate DNA uptake under physiological circumstances. This might actually be a driving factor in autoimmune disease like psoriasis, where LL-37 forms a complex with self-DNA and activates pDCs [91]. 

DNA- and RNA-mediated TLR activation in pDCs and macrophages is a common feature of cathelicidins and has been demonstrated for among others eCATH-2, BMAP-27, BMAP-28, BMAP-34, Bac-1, PMAP-23, PMAP-36 and PR-39 [26,66,92]. Porcine cathelicidins PMAP-23, PMAP-36 and protegrin 1 can potentiate the uptake of nucleotides in various forms, such as plasmid DNA, CpG DNA, genomic DNA, or RNA [66]. Additionally, both LL-37 and mCRAMP can enhance TLR3 signaling in a human lung cell line; but only LL-37 mediates this effect through FPRL1 [93]. 

The capacity of cathelicidins to stimulate TLR3 via dsRNA, TLR7, and TLR8 via ssRNA and TLR9 via dsDNA might be used in vaccine development against bacterial and viral diseases [72,94]. LL-37 does not stimulate TLR9 activation in B cells and pDCs when incubated with human DNA, which makes it suitable as a vaccine adjuvant [90]. However, it is important to investigate whether administration of exogenous cathelicidins does not elicit auto-immune reactions. 

### 3.3. Anti-Inflammatory Immune Modulation

#### 3.3.1. Inhibition of Endotoxin-Mediated TLR Activation

Bacterial endotoxins, such as LPS, lipoteichoic acid (LTA), and flagellin are activators of TLR4, TLR2, and TLR5, respectively [95]. Systemic immune activation by endotoxins can be fatal, so inhibition of inflammatory reactions during sepsis is essential to protect the host from immune overactivation. 

**Figure 3:**
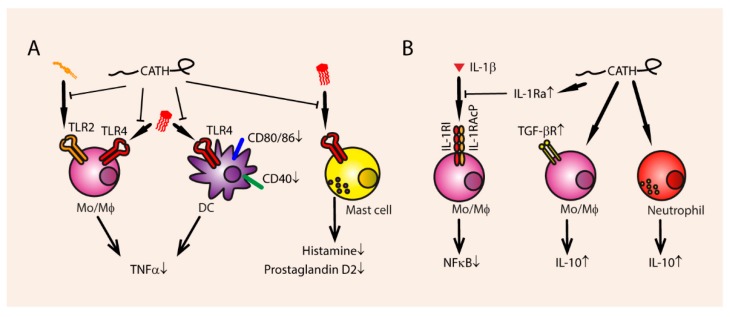
Inhibition of endotoxin-mediated TLR activation by cathelicidins. (**A**) Cathelicidins can bind to LPS, preventing TLR4 activation and thereby reducing TNFα production in monocytes, macrophages and dendritic cells (DCs), also reducing upregulation of CD80/86 and CD40 in DCs. This TLR4 blocking activity can also result in decreased release of histamine and prostaglandin D2 by mast cells. Additionally, cathelicidins can bind the TLR2 ligand LTA, thereby preventing TNFα secretion by monocytes. (**B**) Cathelicidins can directly upregulate IL-10 production in monocytes and neutrophils, and induce upregulation of TGF-βR. Additionally, the upregulation of the IL1β antagonist IL-1Ra means that IL-1β induced NF-κB activation can be reduced.

Cathelicidins can dampen endotoxin-mediated immune reactions by binding the endotoxin and preventing TLR signaling (Figure 3). Human LL-37, rabbit Cap-18, bovine Bac7, BMAP-28, ovine SMAP-29, and chicken chCATH-2 are able to bind directly to negatively charged LPS from Gram-negative bacteria [29,46,73,74,96,97,98,99]. Binding to TLR4 ligands means that many cathelicidins, such as LL-37, mCRAMP, dog K9, PMAP-36, BMAP-27, BMAP-28, indolicidin, SMAP-29, and chCATH-1, chCATH-2, and chCATH-3 can inhibit LPS-induced TNFα production in different leukocytes, such as macrophages, monocytes, and DCs [26,29,97,100,101,102]. 

Furthermore, LTA-induced TNFα production is inhibited by eCATH-2 and PMAP-23, but not K9CATH (Figure 3) [26,46,79]. Interestingly, TLR2 activation by LTA induces LL-37 expression in human macrophages, which can in turn reduce the other LTA-induced inflammatory effects like TNFα and IL-6 production [103]. 

Indolicidin and LL-37 inhibit LPS induced TNFα production at physiological concentrations of 1 to 5 µg/mL [100,101]. Surprisingly, indolicidin, bMAP-27, and LL-37 inhibit LPS-induced TNFα production even when administered 1 h after LPS exposure, but to a lower extent than co-administration [79,100,104]. Thus, inhibition of LPS-mediated TLR4 activation is not solely due to the binding of cathelicidins to LPS, but possibly also due to modification of cellular processes and/or epigenetics. 

LL-37 and BMAP-27 can inhibit endotoxin-induced NFκB translocation to the nucleus in monocytes by increasing the amount of IκBα and by stimulating TNFα-induced protein 3 (TNFAIP-3), which has anti-inflammatory capacities [101,104]. BMAP-28 inhibits not only TLR4 activation but also its internalization, which blocks its ability to stimulate TRAF, TRIF, and IRF3 activation, resulting in less IFN-β expression [73].

Furthermore, cathelicidins inhibit endotoxin mediated up-regulation of other pro-inflammatory cytokines, such as IL-12, IL-8, IL-6, IL-1β, and IFNγ [28,73,79,102,105,106]. mCRAMP inhibits LPS-induced up-regulation of co-stimulatory molecules, such as CD40, CD80, and CD86 on DCs [106]. LPS-induced macrophage activation and mast cell degranulation are also inhibited by LL-37 and rabbit CAP18 [61,98,107]. The presence of LPS inhibits the ability of LL-37 to induce Th1 responses in DCs [108]. Thus, LL-37 does not only block LPS-mediated pro-inflammatory reactions, but LPS also blocks LL-37-mediated immune modulations. LL-37 and LPS can bind through electrostatic interactions and therefore possibly block each other’s capabilities to stimulate cellular processes [109]. The negatively charged, hydrophobic LPS molecules are a suitable binding partner for the cationic, amphipathic cathelicidins, although binding affinity does not necessarily indicate receptor inhibition [105]. 

In vivo, the biological function of cathelicidins released by neutrophils could be that a local high concentration of peptides directly kills bacteria, while simultaneously neutralizing endotoxins released from killed bacteria and thereby preventing excessive immune activation. Indeed, chCATH-2 and PMAP-36 can both kill *E. coli* and prevent immune activation under physiological conditions. LL-37, mCRAMP, chCATH1- and 3, K9CATH, and eCATH-2 can only inhibit immune activation when the bacteria are non-viable [75]. This points to a function for cathelicidins as immunomodulators rather than bactericidal effector molecules.

#### 3.3.2. Secretion of Anti-Inflammatory Cytokines

Besides inhibiting endotoxin-mediated pro-inflammatory cytokine production, cathelicidins stimulate the production of anti-inflammatory cytokines (Figure 3). chCATH-2 upregulates IL-10 in chicken PBMCs [110]. LL-37 upregulates anti-inflammatory receptors such as TGF-βR, and stimulates the secretion of the anti-inflammatory cytokine IL-10 [79]. LL-37 also simulates the release of IL-1Ra in neutrophils, an IL-1β antagonist [111], and inhibits IL-1β and TNFα production by IL-32 induced inflammatory monocytes [112]. At 5 µg/mL, no IL-10 induction was mediated by LL-37 in isolated neutrophils or total PBMCs [81]. Therefore, it is unclear whether cathelicidins can increase expression of anti-inflammatory molecules sufficiently under physiological circumstances to elicit an effect in vivo. Rather, the neutralization of endotoxin seems to be the most relevant mode of anti-inflammatory action.

The systemic anti-inflammatory effects of cathelicidins are clearly demonstrated in in vivo infection models. Bovine BMAP-28 (2 mg/kg) reduced sepsis mortality in mice when injected intravenously with *S. aureus*, to the same levels as the antibiotic imipenem (7 mg/kg) [97]. Lethality was reduced from 100% (control) to 30%. TNFα and IL-6 levels were also significantly reduced in the mice. Intraperitoneal administration of bovine Bac7 (1 mg/kg), and ovine sMAP-29 also reduced septic mortality in rats, induced by intraperitoneal administration of live *E. coli*, to the same levels as the antibiotic polymyxin B (1 mg/kg), reducing mortality from 100% to 20% for sMAP-29 and 27% for Bac7 [96,113]. However, i.p. injection of both bacteria and peptide means the bacterial load could be lower due to direct bactericidal action of a locally high concentration of peptide. Endogenous mCRAMP production is induced by *Clostridium difficile*, but fails to protect the host from inflammatory damage, whereas adding exogenous LL-37 or mCRAMP reduces TNFα production induced by toxins [114]. However, in a polymicrobial sepsis model, mCRAMP knockout mice had increased survival compared to the wild-type [115]. 

The anti-inflammatory effects of cathelicidins can also be seen in some immune diseases. Mice that lack mCRAMP showed more severe contact dermatitis responses than control mice [106]. Administration of intravenous mCRAMP (4 mg/kg) reduced the severity of the inflammation in the mCRAMP-negative mice. mCRAMP negative mice also show more severe immune reactions during acute pancreatitis, having more TNFα production and tissue damage than wild type littermates [116]. 

These findings suggest that cathelicidin-derived compounds could provide a promising new therapy against severe infections, such as sepsis and immune diseases.

### 3.4. Chemotaxis

**Figure 4:**
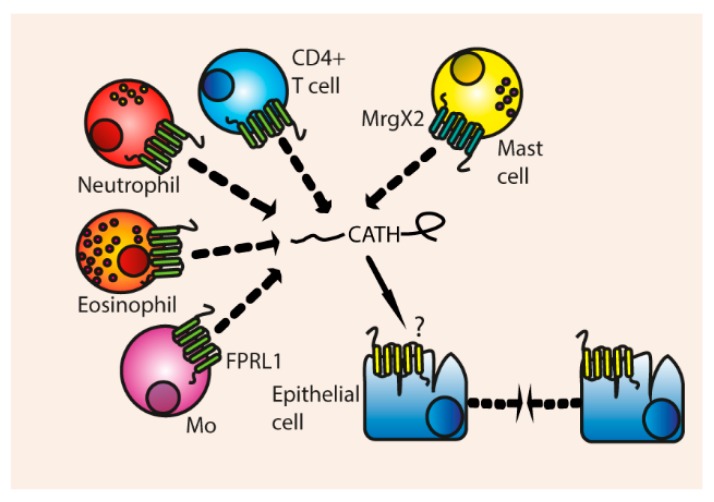
Direct induction of chemotaxis and cellular migration. Cathelicidins induce migration of T cells, neutrophils, eosinophils and monocytes through the FPRL1 receptor. Mast cell chemotaxis is induced through the MrgX2 receptor. Additionally, epithelial cells are induced to migrate through unknown receptors.

Cathelicidins can bind directly to chemoattractant receptors on immune cells and induce migration (Figure 4). Neutrophils are one of the cells responding to infection, and degranulation leads to a release of large amounts of cathelicidins. Therefore, a high local concentration of cathelicidins is found in inflammatory sites.

Human LL-37 has been shown to attract a variety of leukocytes, such as neutrophils, eosinophils [117], monocytes and CD4^+^ T cells via the FPRL1 receptor [118], and attracts mast cells via their MrgX2 receptors, resulting in ERK phosphorylation and Ca^2+^ mobilization [63,119]. LL-37 does not attract monocyte-derived immature DCs, because they downregulate FPRL1 during differentiation [120]. The chemotactic ability of LL-37 is not hampered by serum, and LL-37 is able to attract monocytes [118], mast cells [119], and induces migration of epithelial cells [121] at physiological concentrations of 5 µg/mL. 

It is remarkable that LL-37 can induce chemotaxis of different cell types via two distinct non-homologous receptors, MrgX2 and FPRL1. Therefore, one might wonder whether LL-37 or other cathelicidins are able to induce migration via other receptors that have not been investigated yet. Besides that, it is possible that some chemotactic effects of LL-37 have not been noticed yet due to use of cell lines, instead of blood isolated leukocytes. For instance, Bowdish et al. were not able to detect monocyte migration with the THP-1 cell line, whilst Yang et al. could observe LL-37-induced migration in blood-derived monocytes [118,122]. 

Cathelicidins from other species can also attract different leukocytes even from different species, although their properties are often not as extensively studied as human LL-37. Mouse mCRAMP has been shown to induce chemotaxis of monocytes, macrophages, and neutrophils via the human FPRL1 and mouse FPR2 receptor. FPRL1 and FPR2 activation results in Ca2+ mobilisation and ERK phosphorylation in monocytes [123]. mCRAMP was also able to attract neutrophils and monocytes in vivo at concentrations of 10 µg/mL. Just like LL-37, mCRAMP is not able to attract monocyte-derived DCs. 

Other cathelicidins that can mediate chemotaxis are bovine bMAP-28 [124] and chicken chCATH-1 [56] which attract neutrophils, bovine Bac2a that attracts monocytes and macrophages [100], Bac7 in its pro-form can attract neutrophils [125] and rat rCRAMP which stimulates mast cells migration [126]. Porcine PR-39 has been shown to stimulate neutrophil migration even at concentrations of 0.5 µg/mL [127]. PR-39 and chCATH-1 were not able to induce migration of mononuclear leukocytes such as monocytes, and alveolar macrophages [56,127]. 

The cathelicidins that are released at a site of infection (mainly by neutrophils) will attract other immune cells, which may then also secrete cathelicidins, resulting in a local high concentration of cathelicidins. This cumulative local concentration might even be high enough for the cathelicidins to be bactericidal, even in the presence of salt. Furthermore, the secreted cathelicidins will attract innate, but also adaptive immune cells and therefore help to induce proper immune responses against pathogens. 

#### Indirect Chemotaxis

**Figure 5:**
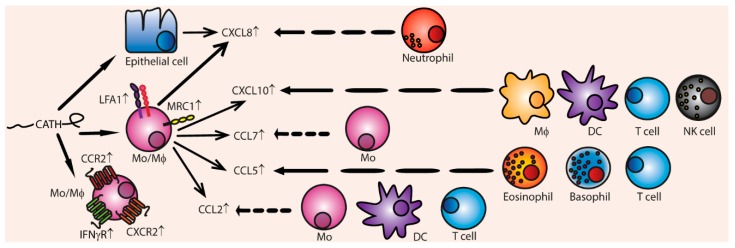
Cathelicidins indirectly induce chemotaxis by inducing the production of various chemokines and chemokines receptors. Cathelicidins can induce chemokine receptor production in monocytes of CCR2, CXCR2, IFNγ-R, MRC1, and LFA1. Additionally, stimulated monocytes produce CCL2, CCL5, CCL7, CXCL10, and CXCL8 (IL-8). Epithelial cells can also be induced to produce CXCL8. The main cell types responding to the chemokines are depicted.

Besides their direct chemotactic functions, cathelicidins can indirectly stimulate chemotaxis by stimulating expression of chemokines and chemokine receptors (Figure 5). CCL2 is up-regulated in various leukocytes by human LL-37 [79,122], PMAP-36 [26], and chCATH-1, chCATH-2, and chCATH-3 [110]. CCL2 especially attracts monocytes, T cells, and DCs towards the site of infection. CCL5, which stimulates chemotaxis of eosinophils, basophils, and T cells, is up-regulated by LL-37, canine K9CATH, and chCATH-2 [26]. CXCL10 expression, which attracts macrophages, T cells, NK cells, and DCs, is induced by LL-37, mouse mCRAMP, canine K9CATH, and equine eCATH-3, [26] IL-8 production is increased by LL-37 [79,117,122], porcine PMAP-23 [46] and PR-39 [74], indolicidin [100], and chicken chCATH-2 [105]. IL-8 stimulates neutrophil migration. Expression of CCL7, also known as MCP-3, is upregulated by LL-37 and chCATH-2 [79,105,122].

Furthermore, 50 µg/mL LL-37 up-regulates the expression of chemokine receptors in mouse macrophages, such as IL-8 receptor, CXCR4, CCR2, and LFA-1 [79]. LL-37 and chCATH-2 also upregulates mannose receptor MRC1 in chicken PBMCs, which is also important for chemotaxis [110].

All in all, cathelicidins can indirectly induce influx of a great variety of innate and adaptive immune cells towards inflammatory sites by modulating chemokine and chemokine receptor expression. Up-regulation of the chemokine genes is induced via activation of EGFR, ERK, and p38 MAPK pathways in in vitro models [122,128]. However, it is not clear whether cathelicidins greatly induce up-regulation of chemokines and chemokine receptors in vivo, since the concentrations cathelicidins that are needed to modify chemokine expression exceed the cathelicidin concentrations that are present in body fluids during infections. For instance, 50 to 100 µg/mL LL-37 was needed to up-regulate expression in IL-8, CCL2 [79], and CCL7 in monocytes [122] and IL-8 in epithelial cells [128]. On the other hand, 10 µg/mL indolicidin or LL-37 can stimulate IL-8 expression in airway epithelial cells and mast cells, but the increase on a protein level is minimal [62,100]. Thus, it is controversial whether IL-8 is up-regulated by cathelicidins during infection, but it is possible that local concentrations of cathelicidins during infections could reach high levels and could induce chemokine expression. 

Thus, cathelicidins can act as direct chemoattractant for a variety of immune cells, and this effect is possibly enhanced by their indirect effect to induce up-regulation of chemokines and chemokine receptors on leukocytes, mediating an influx of immune cells to the site of infection. 

### 3.5. Cell Differentiation and Proliferation

**Figure 6:**
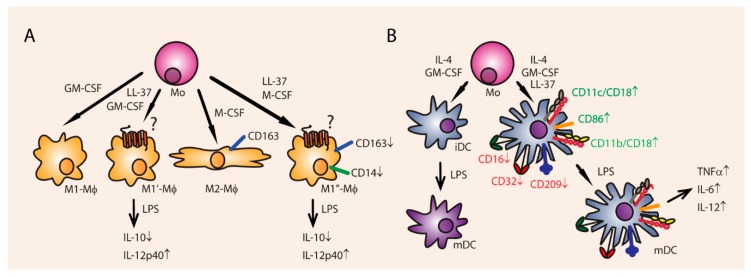
LL-37 effects on cell differentiation. (**A**) Exposure of M2 macrophages to LL-37 polarizes them into a more inflammatory (M1) phenotype, leading to a downregulation of CD163 and CD14 and reduced secretion of IL-10. Additionally, already polarized M1 macrophages produce even more IL-12p40 when co-incubated with LL-37. (**B**) Human blood monocyte-derived DCs also become more inflammatory when exposed to LL-37, leading to a downregulation of CD16, CD32, and CD209, upregulation of CD11c/CD18, CD11b/CD18 (MAC-1), and co-stimulatory CD86. After maturation by LPS stimulation, the LL-37 exposed DCs could produce more TNFα, IL-6, and IL-12.

Cathelicidins can also modify immune responses on a broader level by influencing differentiation of immune cells and by enhancing expression of receptors in antigen presenting cells (APCs). 5 µg/mL of LL-37 is able to increase blood monocyte polarization of macrophages towards the pro-inflammatory M1 type (Figure 6) [129]. Administration of 10 µg/mL LL-37 for 6 days to fully matured anti-inflammatory M2 macrophages even resulted in enhanced M1 cytokine secretion such as IL-12p40, and diminished secretion of M2 cytokines such as IL-10 in the M2 macrophages [129]. LL-37 needs to be endocytosed before it can alter differentiation, but it is not known via which receptors it mediates its influence.

Besides that, cathelicidins influence the function and differentiation of DCs, and are therefore able to steer both the innate and adaptive immune responses. First of all, LL-37 stimulates the differentiation of monocytes towards immature DCs (iDCs) when administered for 24 h at 50 µg/mL [130]. FPR2 signaling (mCRAMP receptor) promotes DC maturation, while FPR2 knockout DCs have impaired maturation in response to LPS [131]. 

Furthermore, cathelicidins stimulate Th1 orientated reactions in DCs (Figure 6). Monocyte derived immature DCs express more HLA-DR and co-stimulatory CD86 when incubated with 30 µg/mL LL-37 for 12 h [132], which was also observed in chicken PBMCs stimulated with LL-37, chCATH-2 [110], and a truncated chCATH-1 analog [56]. Furthermore, LL-37 enhances the endocytic capacity of iDCs, and up-regulates phagocytic receptor expression, such as Fcy receptors CD16 and CD32, complement receptor CD11b/CD18 and CD11c/CD18 [130]. Besides that, LL-37 stimulates the production of co-stimulatory molecules and secretion of Th1 inducing cytokines in human monocyte derived DCs, such IL-12, IL-6, and TNFα [130]. It is not known how LL-37 induces these effects in DCs, but it appears that LL-37 is localized inside the nucleus after it has been endocytosed [132]. Since LL-37 can bind directly to RNA and DNA it is possible that LL-37 itself acts directly as a transcription factor or enhancer [72].

Relating to this Th1-stimulating effect in DCs, there was an abundance of IgG1 antibody producing B cells and IL-4 producing CD4^+^ T cells in mCRAMP KO mice, indicating a negative regulatory role for mCRAMP in type 2 immune response [133]. Not much is known about Th17-related differentiation. LL-37 is found in elevated concentration in Th1/Th17 associated autoimmune skin diseases [134,135], and can be a T-cell auto-antigen in psoriasis [136]. 

**Figure 7:**
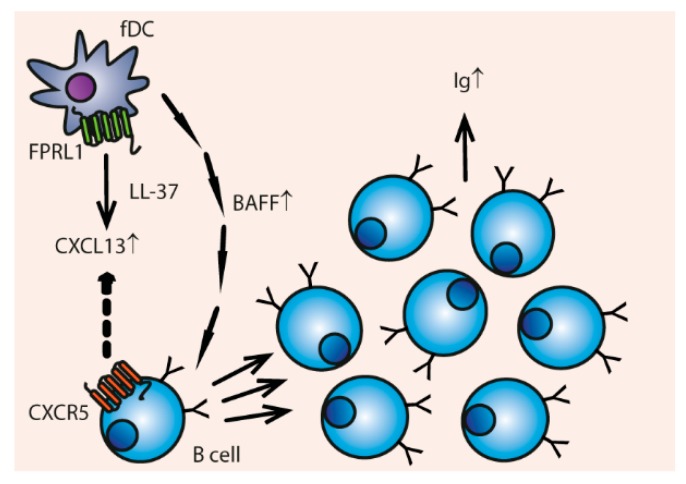
LL-37 induces B-cell attracting chemokines and enhances B-cell proliferation. Follicular DCs (fDCs) produce CXCL13 through LL-37-induced FPRL1 signaling; this chemokine attracts B cells through the CXCR5 receptor. Additionally, the fDCs produce more B cell-activating factor (BAFF), which enhances B cell proliferation and IgA production.

Besides monocyte-derived DCs, LL-37 influences follicular DCs (fDCs) (Figure 7). LL-37 activates the FPRL1 receptor on fDCs, which results in up-regulation of CXCL13 that attracts B cells via their CXCR5 receptor [137,138]. The production of B cell activating factor (BAFF) is also enhanced by LL-37. This leads to increased B cell proliferation and immunoglobulin secretion [137].

Most of the pro-Th1 induced functions of cathelicidins are hampered in the presence of LPS. LL-37 together with LPS does not elicit IL-6 and TNFα production by DCs and they do not up-regulate CCR7, CD86 or HLA-DR. Additionally, co-cultured T cells proliferate less and produce less IL-2 and IFNy [108]. Thus, LL-37 and LPS alone can both stimulate Th1 skewing responses in DCs, but when added together inhibit Th1 responses [108]. This is probably due to the endotoxin-neutralizing properties of LL-37. The same interaction is seen between LL-37 in combination with LTA or flagellin [108].

Besides that, administration of LPS together with LL-37 inhibits IgM and IgG2a production in B cells, B cell proliferation, and class switching [102]. LPS alone stimulates IgG2a antibody production in B cells. Again, the pro-Th1 effect of LL-37 and LPS alone seem to be neutralized when the two components are added together. 

Since cathelicidins can stimulate Th1 reactions in the absence of LPS, they could be used as adjuvants in vaccines to boost adaptive immune responses. When mice were immunized with ovalbumin and chicken chCATH-1 and received a booster at day 14, the mice produced higher levels of IgG1 and IgG2a when challenged with ovalbumin on day 20 [56]. Remarkably, when mouse mCRAMP was administered to mice together with ovalbumin as antigen it induced up-regulation of IgG1, IgG2a, IgG2b, and IgG3, and stimulated production of IFNγ and IL-4 [123]. Thus, the immune reaction after mCRAMP stimulation did not show a specific polarization towards Th1, but rather consisted of both Th1 and Th2 responses. 

Besides polarizing immune reactions, the DNA and RNA binding and subsequent TLR3, TLR7, TLR8 and TLR9-activating properties of cathelicidins can make cathelicidins suitable adjuvants [26]. It is important to consider that presence of other TLR ligands, such as LPS or LTA in the vaccine can change the effects of cathelicidins on the induction of Th1 responses [108]. If this modulatory effect of cathelicidins can be harnessed to influence specific type of immune response desired by the vaccine, cathelicidins could be very useful adjuvants. 

## 4. Conclusions

Cathelicidins have a wide range of functions on many different cell types, but due to their pleiotropic effects therapeutic use also has its limitations. For instance, they have both pro-tumorigenic and anti-cancer effects, which depend on the tumor type [139]. Cathelicidin-derived compounds could be applicable in many kinds of therapies including pro-inflammatory in the case of vaccines or anti-inflammatory in the case of sepsis, but care must be taken to avoid tumor- or auto-immune-inducing side effects.

From a therapeutic point of view, a marked difference exists between administering exogenous cathelicidins and inducing endogenous production. Stimulation of endogenous production will likely have fewer unforeseen side effects but is limited to the host’s repertoire. For humans, who carry only one cathelicidin gene, this approach seems rather limited. Conversely, exogenous peptides can have any origin (including non-natural sources like in silico design), but will likely be more transient, and have less target-specific effects. Indeed, the specific effects of a cathelicidin are highly dependent on which cathelicidin is used in which concentration, the environmental parameters like salt concentration and inflammatory context, and which cell type is studied. Therefore, it is important to continue testing cathelicidins from diverse origins in different experimental setups. 

Currently, the most efficient way of producing cathelicidins is to use traditional peptide synthesizers. This gives a lot of control over the sequence and allows use of unconventional amino acids. Given their antimicrobial potential, recombinant expression in bacterial species is a challenge. Some methods like bacterial production of fusion proteins or inteins do exist, for example in *E. coli* [140], *Bacillus subtilis* [141], or the yeast *Pichia pastoris* [142]. The purified peptides often retain their antibiotic activity but are (in these examples) not tested for endotoxins or immunomodulatory activity. The LPS-binding properties of cathelicidins makes purification of endotoxin-free solution especially challenging. Therefore, other recombinant expression systems might be more applicable, like plants or cell lines. Cathelicidins successfully expressed in plants include LL-37 and SMAP-29 [143,144]. 

After exogenous in vivo administration, unmodified peptide is degraded readily by both host and microbial factors [64,145,146]. If the therapeutic goal requires longer exposure time to the peptides, either multiple administrations are needed, or a slow release system. Another possibility is to protect the mature peptide from degradation, for example by using unusual amino acids, D-enantiomers, or point mutations to limit the peptides’ susceptibility to proteolytic degradation. 

For therapeutic use as antimicrobials, immobilized cathelicidins are a good avenue to pursue. There are examples of immobilized peptides working effectively at preventing bacterial outgrowth [54,147]. Immobilized peptides could lose some antibacterial activity depending on their length and orientation, but can also be more resistant to degradation [147]. There are multiple chemical methods of coupling peptides using specific residues [148]. In short, whereas direct (for example intravenous) application of cathelicidins as antimicrobials is probably not a very effective treatment, prevention of biofilms growing on medical devices can give major health benefits, since indwelling medical devices are a major source of nosocomial infections [149]. Additionally, since cathelicidins stimulate wound healing [107,150,151] and angiogenesis [107,152,153], they could be used as treatment supplementation for wounds at risk of infection like surgical wounds. 

As immunomodulators, cathelicidins could also be used as vaccine adjuvants or additives. Their small size means they can easily be incorporated in already existing formulations. They attract immune cells to the site of injection and polarize DCs and T-cells to specific types of immune reaction. Depending on the vaccine, this could be an answer for specific therapeutic questions. Cathelicidins could also be suitable as immunopotentiators on next-generation nanoparticle vaccines [154]. However, not much is known on immunomodulation by immobilized cathelicidins. 

In conclusion, cathelicidins are expressed in many different forms by most animals and have varied immunomodulatory functions. The specific effects are highly dependent on the specific cathelicidin, the concentration, presence of other factors, and the inflammatory context. This diversity means they could potentially be applicable in a wide variety of immunomodulatory therapies.
